# Tailoring low-dose aspirin to prevent preeclampsia: translational and biomarker insights

**DOI:** 10.3389/fmed.2026.1754660

**Published:** 2026-02-18

**Authors:** Hira Sohail, Xiao Lin Hua

**Affiliations:** Shanghai First Maternity and Infant Hospital, School of Medicine, Tongji University, Shanghai, China

**Keywords:** angiogenic biomarkers, fetal growth restriction (FGR), genetic, low dose aspirin, platelet, preeclampsia, pregnancy

## Abstract

**Background:**

Low-dose aspirin is the only pharmacological intervention with consistent evidence for reducing the risk of preeclampsia in high-risk pregnancies. However, substantial interindividual variability in response has prompted interest in biomarkers that may improve understanding of aspirin responsiveness and disease heterogeneity.

**Methods:**

This narrative review synthesizes translational, observational, and clinical literature examining genetic, platelet, and angiogenic biomarkers in the context of aspirin prophylaxis for preeclampsia. Relevant studies were identified through targeted searches of major biomedical databases to provide a conceptual overview of current evidence.

**Results:**

Genetic variants related to aspirin metabolism and platelet function, platelet indices and aggregation assays, and angiogenic biomarkers such as soluble fms-like tyrosine kinase-1 and placental growth factor have been investigated as potential tools to refine risk stratification and elucidate variability in aspirin response. While these biomarkers offer important mechanistic insight, most evidence derives from association studies and observational cohorts. Genetic testing and platelet function assays lack validation in pregnant populations, standardized thresholds, and randomized evidence, supporting their use to guide aspirin initiation, dosing, or monitoring. Angiogenic biomarkers have an established diagnostic and prognostic role later in pregnancy but remain investigational for first-trimester risk stratification and are not used to modify aspirin therapy.

**Conclusions:**

Biomarkers provide valuable insight into the biological heterogeneity underlying preeclampsia and aspirin response; however, biomarker-guided aspirin strategies remain investigational. In the absence of randomized trials, aspirin prophylaxis should continue to follow established guideline-based risk assessment, with biomarker-informed approaches reserved for future research.

## 1 Introduction

Preeclampsia (PE) is one of the most significant hypertensive conditions unique to pregnancy, affecting roughly 2%−8% of pregnancies worldwide (1). It remains a major driver of maternal death, serious complications, early delivery, fetal growth restriction, and long-term cardiovascular risk for both mothers and their children (2). Despite extensive research, PE continues to pose difficulties for clinicians because its onset is hard to predict, its symptoms vary widely, and its biological mechanisms are multifactorial (3). Traditionally, PE is diagnosed when a pregnant individual develops new hypertension after 20 weeks of gestation, along with proteinuria or evidence of maternal organ dysfunction (4). Increasingly, however, it is viewed as a syndrome encompassing several distinct subtypes, such as early-onset and late-onset forms, as well as placental, immune-related, and metabolic phenotypes (5).

The prevailing model describes PE as developing through two major stages (6). The first involves inadequate trophoblast invasion and poor remodeling of the uterine spiral arteries, which leads to defective placentation and persistent placental hypoxia (6). The second stage reflects the maternal response to this placental stress—characterized by endothelial dysfunction, oxidative stress, exaggerated inflammatory activity, and imbalanced angiogenic signaling (6, 7). These disturbances culminate in the clinical features of hypertension and multi-organ involvement. Even with this framework, PE remains highly diverse, influenced by maternal genetics, immune adaptation, cardiometabolic health, and environmental factors (1).

Since the condition can only be resolved after delivery of the placenta, prevention is essential. Low-dose aspirin (LDA) is currently the only pharmacological measure with consistent evidence for lowering PE risk in individuals considered high-risk (8). However, many eligible patients either show limited benefit or still develop PE despite prophylaxis (9–12). Growing research suggests that maternal genetic variations, platelet activity profiles, and angiogenic biomarkers may affect both predisposition to PE and responsiveness to LDA (13).

This article is a narrative review synthesizing translational, observational, and mechanistic studies; it is not a systematic review. Literature was identified through targeted searches of PubMed and EMBASE focusing on aspirin, preeclampsia, genetic, platelet, and angiogenic biomarkers.

## 2 Low-dose aspirin in the prevention of preeclampsia

Low-dose aspirin (LDA) is currently the most effective and widely recommended pharmacologic strategy to decrease the risk of preeclampsia, particularly forms that develop early in pregnancy (14). Its primary action involves the selective, irreversible inhibition of platelet cyclooxygenase-1 (COX-1), which lowers the production of thromboxane A_2_ (TxA_2_)—a molecule that promotes vasoconstriction and platelet aggregation. At low doses, aspirin spares endothelial cyclooxygenase activity, allowing continued production of prostacyclin (PGI_2_), a vasodilator that counteracts platelet activation (15). By restoring the TxA_2_/PGI_2_ balance, LDA supports healthier uteroplacental blood flow (15).

Clinical trials and meta-analyses consistently support its benefit (15–17). The ASPRE trial demonstrated that starting 150 mg of aspirin before 16 weeks' gestation in women identified as high risk reduced the incidence of early-onset PE by more than 60% (18). Earlier pooled analysis reported an overall reduction of roughly 10%−20% in PE onset, with the most pronounced benefit occurring when treatment begins between 11 and 16 weeks, coinciding with the critical period of spiral artery remodeling (19). This suggests that aspirin may exert its primary protective effect by influencing placental development and attenuating inflammation before irreversible pathology occurs (20).

Despite strong evidence for its use, several issues complicate optimal implementation. International organizations offer differing recommendations: ACOG advises 81 mg daily, whereas FIGO and NICE suggest doses between 100 and 150 mg (21). Additionally, the standard single-dose approach does not address the considerable variability in how individuals process and respond to aspirin. Factors such as maternal body mass index, platelet turnover, genetic variants affecting aspirin metabolism, and preexisting platelet hyperactivity can all alter the degree of COX-1 inhibition achieved (22).

Such variability contributes to the problem of “aspirin resistance,” observed in up to one-third of high-risk pregnancies (23). Persistent platelet activity despite LDA use is associated with a heightened risk of PE, implying that fixed dosing may not be appropriate for all patients. Higher doses—typically 100–150 mg—have been proposed for individuals with elevated BMI or increased platelet activation, yet these strategies are not consistently incorporated into clinical guidelines (24).

Another major challenge is accurately identifying who will benefit most from prophylaxis. Current recommendations are based largely on clinical risk factors such as prior PE, chronic hypertension, diabetes, or multifetal gestation (25). Although useful, these criteria have limited predictive accuracy: many individuals who develop PE are not classified as high risk, while some who are deemed high risk never develop the disease. Incorporating biomarkers—particularly angiogenic markers and platelet function assays—may strengthen prediction models and support more targeted aspirin use (26). In summary, while LDA remains central to PE prevention, its effectiveness is constrained by uniform dosing approaches and broad inclusion criteria. Integrating genetic, biochemical, and physiological markers into clinical decision-making could refine aspirin prophylaxis and enable a shift toward personalized prevention strategies (27). However, no randomized trials have demonstrated improved outcomes using biomarker-guided aspirin dosing in pregnancy.

## 3 Genetic influences on preeclampsia and response to aspirin

Genetic variation plays a significant role in both the development of preeclampsia (PE) and the differing responses to low-dose aspirin among pregnant individuals. PE is known to have a heritable component, with contributions from maternal, fetal, and paternal genomes. Large genomic studies, such as GWAS, have identified numerous loci linked to angiogenic signaling, immune modulation, endothelial function, and placental biology (28). Variants in genes such as FLT1, HLA-G, ENG, ACVR2A, and FGG have been associated with impaired placental vascularization, increased inflammatory activity, and disruptions in immune tolerance—key elements of PE pathogenesis (29, 30).

Genetic differences may also influence how effectively aspirin prevents PE. Since aspirin works through irreversible COX-1 inhibition, polymorphisms in the PTGS1 gene can reduce platelet sensitivity to aspirin and lead to incomplete thromboxane suppression (31). Variants in platelet receptor genes, such as ITGA2 and GP1BA, may heighten baseline platelet activity, meaning higher or earlier dosing could be needed to achieve adequate inhibition (32).

Polymorphisms in enzymes such as CES1 and PON1, which help hydrolyze aspirin, can modulate how quickly the drug is converted into salicylic acid, thereby influencing systemic exposure and effectiveness (33). Genetic differences in endothelial and oxidative stress pathways—for example, NOS3 variants—may further modify aspirin's impact on vascular function (33).

Growing research suggests that genetics may help determine which patients benefit most from LDA. Certain FLT1 or HLA-G alleles are associated with unique angiogenic patterns and distinct PE risk profiles, which may alter responsiveness to antiplatelet therapy (34). Although routine genetic testing is not yet clinically recommended, incorporating polygenic risk scores, platelet-related genotypes, and placental gene expression signatures may ultimately enhance risk stratification. These findings support further investigation into genetically informed prevention strategies; however, routine genetic testing to guide aspirin use is not currently recommended and remains a research priority (35).

## 4 Platelet function biomarkers and their role in aspirin responsiveness

Platelet activation plays a key role in the development of preeclampsia (PE) and contributes to interindividual variability in response to low-dose aspirin (LDA) (22). In PE, heightened platelet reactivity—driven by increased thromboxane A_2_ production, systemic inflammation, and endothelial dysfunction—promotes vasoconstriction, impaired placental perfusion, and activation of coagulation pathways (15). Platelet-related biomarkers, therefore, provide mechanistic insight into PE pathophysiology and aspirin pharmacodynamics; however, their role in guiding clinical management during pregnancy remains investigational (22).

### 4.1 Thromboxane metabolites

Urinary 11-dehydro-thromboxane B_2_ (11-dh-TXB_2_) is a specific marker of aspirin-mediated platelet cyclooxygenase-1 (COX-1) inhibition (36). Elevated levels during prophylaxis reflect incomplete suppression of thromboxane synthesis and have been associated with an increased risk of PE (37). Observational studies suggest that individuals with obesity, chronic hypertension, or genetic variants affecting COX-1 activity may exhibit persistently elevated 11-dh-TXB_2_ levels despite standard LDA dosing (37–39). While these findings highlight variability in aspirin responsiveness, they have not been prospectively validated in pregnancy and do not currently support dose adjustment based on thromboxane metabolite measurements.

### 4.2 Mean platelet volume (MPV) and platelet distribution width (PDW)

Mean platelet volume (MPV) and platelet distribution width (PDW) reflect platelet size and heterogeneity, both of which increase in states of enhanced platelet activation (40). Elevated MPV early in pregnancy has been associated with an increased risk of subsequent PE in observational studies (41). Although altered platelet indices may reflect heightened platelet turnover and reduced sensitivity to aspirin's antiplatelet effects, MPV and PDW are influenced by physiological changes during pregnancy, lack standardized cut-off values, and have not been validated to guide aspirin dosing or monitoring in pregnant populations (42, 43).

### 4.3 Platelet aggregation assays

Platelet aggregation assays, such as light transmission aggregometry (LTA) and the VerifyNow Aspirin assay, provide direct measures of aspirin-induced platelet inhibition (14, 44). While these tools are not part of routine obstetric practice, they are commonly used in cardiovascular medicine to assess aspirin responsiveness (14). In pregnancy, however, their clinical utility remains uncertain due to limited validation, variability in platelet physiology, and the absence of randomized trials, demonstrating improved outcomes when aspirin therapy is guided by aggregation testing.

### 4.4 Platelet-derived microparticles (PMPs)

Platelet-derived microparticles (PMPs) reflect platelet activation and turnover, both of which are increased in PE. Elevated PMP concentrations may indicate heightened platelet activation and rapid platelet renewal, potentially limiting sustained platelet inhibition by aspirin (44). This mechanism has been proposed as a contributor to reduced aspirin responsiveness in individuals with elevated body mass index; however, PMP measurement remains a research tool and is not currently applicable to clinical decision-making in pregnancy.

### 4.5 Limitations and clinical implications

Collectively, platelet biomarkers underscore substantial interindividual variability in aspirin pharmacodynamics during pregnancy. While these markers provide valuable insight into biological mechanisms underlying aspirin response and PE risk, they are influenced by normal gestational changes, lack standardized thresholds, and have not been validated to guide aspirin initiation, dosing, or monitoring. Importantly, no randomized controlled trials have evaluated platelet biomarker-guided aspirin strategies in pregnancy. As such, platelet function testing should not be used to tailor aspirin therapy outside of research settings, and aspirin prophylaxis should continue to follow established guideline-based recommendations (45).

## 5 Angiogenic biomarkers and their role in aspirin-based prevention

Disruption of angiogenic balance is a central feature of preeclampsia (PE), making angiogenic biomarkers—particularly soluble fms-like tyrosine kinase-1 (sFlt-1) and placental growth factor (PlGF)—important tools for understanding placental dysfunction. sFlt-1 acts as a decoy receptor that reduces circulating vascular endothelial growth factor (VEGF) and PlGF, impairing angiogenic signaling and contributing to widespread endothelial dysfunction (46). In contrast, PlGF supports placental vascular development and maternal cardiovascular adaptation. The sFlt-1/PlGF ratio is an established marker of placental health and is widely used for diagnosis and prognosis of PE later in pregnancy, particularly for predicting early-onset disease (46, 47).

### 5.1 Investigational use of angiogenic biomarkers for early risk stratification

Observational studies have demonstrated that elevations in sFlt-1 and reductions in PlGF can precede the clinical onset of PE by weeks to months, especially in individuals at risk for severe, early-onset disease (46–48). Low first-trimester PlGF levels are associated with impaired spiral artery remodeling and early placental dysfunction (46–48). These findings have prompted an investigation into the potential role of first-trimester angiogenic profiling as a research tool for early risk stratification. However, this application remains investigational and has not been validated to guide preventive interventions.

### 5.2 Angiogenic biomarkers and aspirin responsiveness

Emerging data suggest that angiogenic profiles may differ among individuals who subsequently develop PE despite aspirin prophylaxis, raising interest in the interaction between aspirin and angiogenic pathways (49). Experimental and observational studies indicate that aspirin initiated early in pregnancy may modestly influence placental angiogenic signaling, potentially improving placental perfusion (50). While combining angiogenic biomarkers with maternal clinical risk factors improves predictive performance in research settings, angiogenic markers are not currently used to initiate, adjust, or escalate aspirin therapy, such as dose modification, beyond guideline-recommended practice (51).

### 5.3 Barriers to clinical translation and current limitations

Despite robust evidence supporting their diagnostic and prognostic value later in pregnancy, routine use of angiogenic biomarkers for early risk stratification faces several barriers, such as cost, assay availability, and limited access in resource-constrained settings. In addition, there is no consensus regarding optimal testing intervals, interpretation thresholds, or clinical actions based on early pregnancy measurements (51, 52). Importantly, no randomized controlled trials have evaluated angiogenic biomarker-guided aspirin initiation or dosing strategies. As such, the integration of angiogenic markers into aspirin-based prevention should be viewed as a future research direction rather than a component of current clinical care (53).

## 6 Integrative personalized prophylaxis: toward a biomarker-guided aspirin strategy

**Figure 1:**
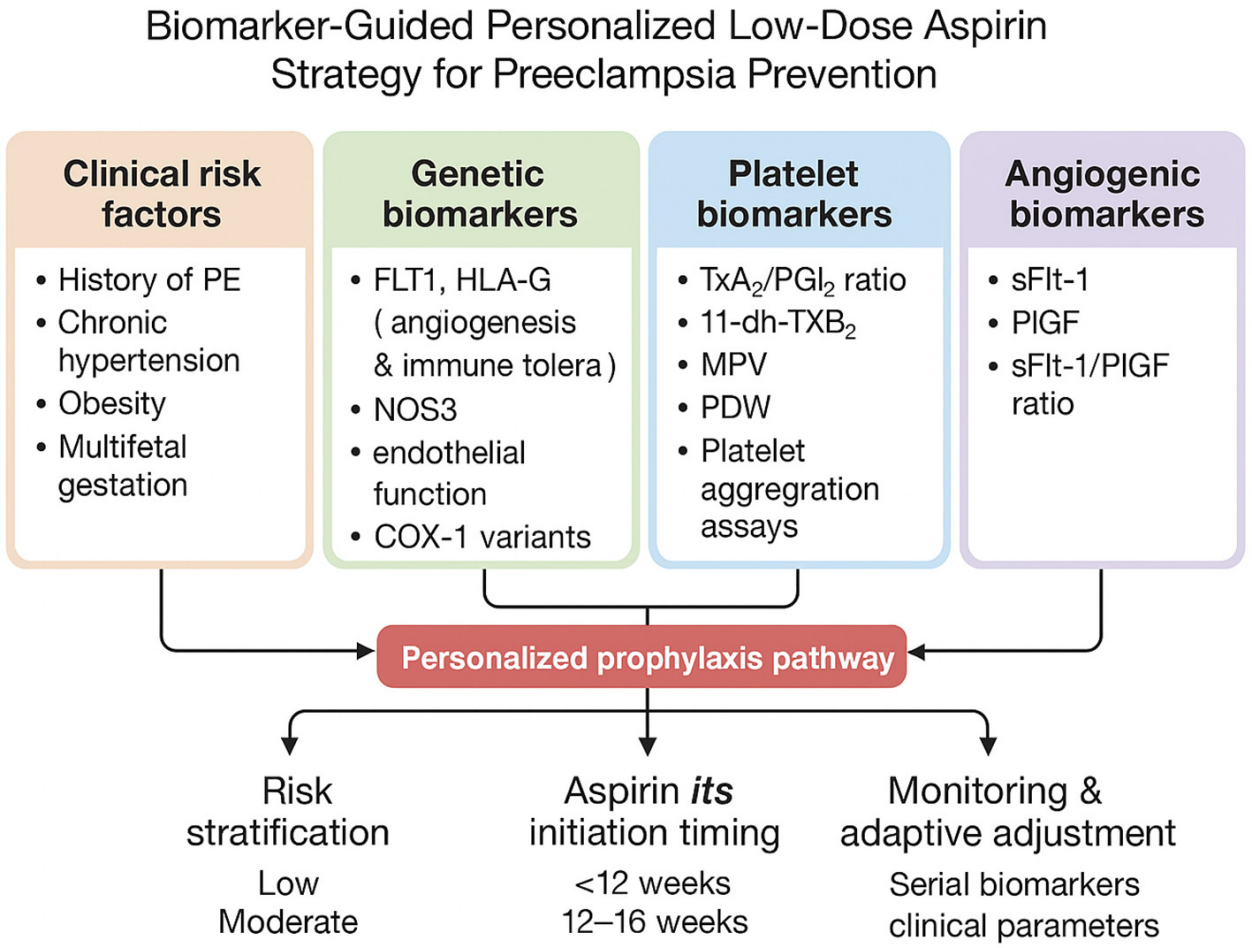
Conceptual framework illustrating the integration of clinical risk factors with genetic, platelet, and angiogenic biomarkers to explore heterogeneity in aspirin responsiveness and preeclampsia risk. This model is intended to highlight potential research directions and hypothesis generation rather than to represent a clinical decision-making algorithm.

Bringing biological insights into clinical practice represents the next major step in optimizing aspirin use for PE prevention. A personalized prevention model combines maternal clinical risk factors with genetic predisposition, platelet function profiles, and angiogenic biomarker data, creating a more dynamic and individualized approach rather than applying a uniform dosing strategy to all patients. Figure 1 provides an integrated conceptual model in which genetic markers (e.g., F2/F5/PTGS1 variants), platelet function indices, and angiogenic biomarkers (such as PlGF and sFlt-1) collectively inform individualized decisions regarding aspirin initiation, dosage adjustment, and monitoring throughout pregnancy.

## 7 Integrative personalized prophylaxis: a conceptual framework for future research

Translating biological insights into improved prevention strategies represents an important goal in preeclampsia (PE) research. Integrative approaches that combine maternal clinical risk factors with genetic, platelet, and angiogenic biomarkers have been proposed as a means of better understanding interindividual variability in aspirin responsiveness. The framework described below is presented as a **conceptual model to illustrate potential future research directions rather than a clinical decision-making algorithm**. Figure 1 depicts this integrative model, in which genetic markers (e.g., F2, F5, PTGS1 variants), platelet function indices, and angiogenic biomarkers (such as PlGF and sFlt-1) are considered alongside clinical risk factors to generate hypotheses regarding heterogeneity in aspirin response.

### 7.1 Multidimensional risk stratification

Current risk assessment for PE relies primarily on clinical factors, such as prior PE, chronic hypertension, obesity, and multifetal gestation (4). While effective at identifying high-risk populations, these criteria lack precision at the individual level. Integrating biomarker data with clinical risk factors may improve understanding of disease heterogeneity and facilitate more refined risk stratification in research settings. For example, observational studies suggest associations between elevated sFlt-1/PlGF ratios, increased thromboxane metabolite levels, or high polygenic risk scores and subsequent PE risk (48, 54, 55). However, these associations remain exploratory and have not been validated to guide aspirin initiation, dosing, or monitoring in clinical practice. Table 1 summarizes proposed biomarker domains and their current level of evidence, highlighting areas for future investigation.

**Table 1:** Summary of maternal risk factors, associated biomarkers, and recommended aspirin strategies for preeclampsia prevention.

**Maternal risk factor**	**Associated biomarkers**	**Evidence type**	**Key findings**	**Current clinical applicability**
Chronic hypertension	sFlt-1/PlGF ratio	Observational, prognostic	Elevated ratios associated with increased risk of early-onset PE	Established for diagnosis/prognosis later in pregnancy; investigational for early risk stratification
Obesity (BMI > 30)	Urinary thromboxane metabolites, MPV	Mechanistic, observational	Increased platelet turnover and reduced aspirin responsiveness observed	Not validated for guiding aspirin dose or timing
Polygenic risk (FLT1 variants)	Polygenic risk scores (FLT1, HLA-G)	Genetic association studies	Certain variants associated with PE susceptibility	Investigational; not recommended for clinical decision-making
Prior preeclampsia	Platelet indices, aggregation assays	Observational	Altered platelet activation reported in recurrent PE	Not validated for aspirin monitoring or dose adjustment
Multiple gestation	sFlt-1/PlGF ratio, platelet biomarkers	Observational	Higher angiogenic imbalance and platelet activation	No evidence to guide aspirin modification beyond guidelines
Elevated platelet activity	11-dh-TXB_2_, platelet aggregation	Mechanistic	Incomplete thromboxane suppression linked to PE risk	Research use only; no randomized trials guiding therapy

### 7.2 Aspirin dose and timing: evidence gaps and research directions

Although current guidelines recommend aspirin doses of 75–150 mg initiated before 16 weeks of gestation, interest has emerged in whether biological heterogeneity may influence optimal dosing or timing (56, 66). Studies exploring genetic risk, angiogenic profiles, platelet turnover, and aspirin pharmacodynamics have generated hypotheses regarding differential responses to aspirin prophylaxis (51). Importantly, no randomized controlled trials have evaluated biomarker-guided aspirin dose adjustment or timing strategies in pregnancy. As such, personalization of aspirin therapy based on biomarker profiles remains investigational and should be considered a priority for future clinical research rather than a component of routine care.

### 7.3 Longitudinal assessment and biomarker monitoring

The concept of dynamic risk assessment across pregnancy has been proposed to better capture evolving placental and maternal vascular changes. In research contexts, longitudinal evaluation of angiogenic markers or platelet-related biomarkers has been used to study disease progression and aspirin responsiveness (49, 51, 54). However, physiological changes during pregnancy, lack of standardized thresholds, and absence of outcome-driven validation currently limit the clinical applicability of serial biomarker monitoring for guiding aspirin therapy.

### 7.4 Toward future clinical translation

Conceptual clinical workflows integrating biomarkers with clinical assessment have been proposed to guide future trial design and translational research. Such frameworks may inform the structure of prospective studies evaluating whether biomarker-informed strategies can improve outcomes compared with standard guideline-based aspirin prophylaxis (57, 58). Until evidence from well-designed randomized trials becomes available, aspirin use for PE prevention should continue to follow established clinical guidelines, with biomarker-guided approaches reserved for research settings.

## 8 Challenges, limitations, and future directions

Personalized aspirin strategies offer significant potential, but several challenges remain to their widespread adoption. A major hurdle is the lack of standardization for biomarkers like sFlt-1, PlGF, and platelet aggregation (54, 55). Assay variability and inconsistent cut-off values across different labs complicate their use in clinical settings. Establishing validated thresholds through large multicenter studies is essential for reliable application in decision-making.

### 8.1 Cost and practical considerations

Advanced biomarker and genomic testing remain costly and may be inaccessible in low-resource settings, despite these regions bearing the highest burden of PE. Widespread adoption of precision obstetrics will require more affordable testing solutions and point-of-care devices. Cost-effectiveness analyses and simplified testing strategies are essential for broad implementation (59).

### 8.2 Biological complexity of PE

PE is a heterogeneous condition, with various underlying pathways, such as angiogenic, immunologic, and metabolic factors. Not all PE subtypes respond to aspirin, meaning some may require additional interventions, such as antioxidants, statins, or endothelial stabilizers (60). A precision approach must recognize that aspirin alone may not suffice, and combined therapies may be needed for optimal prevention.

### 8.3 Timing limitations and clinical constraints

While aspirin is most effective when started before 12–16 weeks, many individuals do not access prenatal care early enough (56). Adherence may also be affected by gastrointestinal discomfort or uncertainty about dosing (61). Timely engagement with prenatal care services and improved patient education are key to overcoming these barriers.

### 8.4 Ethical considerations and data protection

Genomic and biomarker testing raises important ethical concerns, particularly around data security, incidental findings, and equitable access (62). A robust framework for informed consent and data protection is crucial for ensuring the ethical implementation of personalized screening programs.

### 8.5 Future research directions

Future research should focus on developing machine learning models that combine genomic, metabolic, and clinical data to refine risk stratification. Advances in affordable, point-of-care angiogenic assays will facilitate widespread clinical use (63). Identifying aspirin-resistant PE phenotypes through metabolomics or platelet profiling, along with randomized trials of genotype- or biomarker-guided dosing, will help optimize aspirin therapy (64). Additionally, exploring adjunct therapies (e.g., pravastatin and metformin) for patients unlikely to respond to aspirin alone could provide a more comprehensive approach to PE prevention (65).

## 9 Conclusion

Low-dose aspirin remains the cornerstone of pharmacological prevention for preeclampsia in pregnancies identified as high risk using established clinical criteria. Growing interest in genetic, platelet, and angiogenic biomarkers reflects recognition of the biological heterogeneity underlying both preeclampsia pathogenesis and variability in aspirin responsiveness. This narrative review highlights the potential of these biomarkers to enhance mechanistic understanding and to inform future research aimed at refining risk stratification and preventive strategies.

However, current evidence supporting biomarker-guided approaches to aspirin prophylaxis is predominantly derived from mechanistic studies, genetic association analyses, and observational cohorts. Genetic profiling and platelet function testing lack validation in pregnant populations, standardized thresholds, and randomized evidence, demonstrating improved maternal or perinatal outcomes. Similarly, while angiogenic biomarkers have an established diagnostic and prognostic role later in pregnancy, their use for first-trimester risk stratification and for guiding aspirin initiation or dose modification remains investigational.

Importantly, no randomized controlled trials have evaluated biomarker-guided aspirin dosing or monitoring strategies in pregnancy. Until such evidence becomes available, the use of aspirin for preeclampsia prevention should continue to follow current guideline-based recommendations regarding patient selection, timing, and dosage. Biomarker-informed strategies should be regarded as a future research direction rather than a component of routine clinical care.

Future studies should prioritize well-designed prospective cohorts and randomized trials to evaluate whether integrating biomarkers with clinical risk assessment can meaningfully improve outcomes without increasing harm. Such evidence will be essential before precision-based approaches to aspirin prophylaxis can be responsibly translated into clinical practice.

## References

[1] Martini C Saeed Z Simeone P Palma S Ricci M Arata A . Preeclampsia: insights into pathophysiological mechanisms and preventive strategies. Am J Prev Cardiol. (2025) 23:101054. doi: 10.1016/j.ajpc.2025.101054 40703703 PMC12284657

[2] Leeson P. Long term cardiovascular outcomes for mother and child. Pregnancy Hypertens Int J Womens Cardiovasc Health. (2013) 3:60–1. doi: 10.1016/j.preghy.2013.04.012 26105846

[3] Ramos JGL Sass N Costa SHM. Preeclampsia. Rev Bras Ginecol Obstet. (2017) 39:496–512. doi: 10.1055/s-0037-1604471 28793357 PMC10309474

[4] Fox R Kitt J Leeson P Aye CYL Lewandowski AJ. Preeclampsia: risk factors, diagnosis, management, and the cardiovascular impact on the offspring. J Clin Med. (2019) 8:1625. doi: 10.3390/jcm8101625 31590294 PMC6832549

[5] Han L da Silva Costa F Perkins A Holland O. Molecular signatures of preeclampsia subtypes determined through integrated weighted gene co-expression network analysis and differential gene expression analysis of placental transcriptomics. Front Cell Dev Biol. (2025) 13:1635878. doi: 10.3389/fcell.2025.1635878 40823529 PMC12350358

[6] Roberts JM Hubel CA. The two stage model of preeclampsia: variations on the theme. Placenta. (2009) 30:32–7. doi: 10.1016/j.placenta.2008.11.009 19070896 PMC2680383

[7] Chiang YT Seow KM Chen KH. The pathophysiological, genetic, and hormonal changes in preeclampsia: a systematic review of the molecular mechanisms. Int J Mol Sci. (2024) 25:4532. doi: 10.3390/ijms25084532 38674114 PMC11050545

[8] Caritis S Sibai B Hauth J Lindheimer MD Klebanoff M Thom E . Low-dose aspirin to prevent preeclampsia in women at high risk. N Engl J Med. (1998) 338:701–5. doi: 10.1056/NEJM199803123381101 9494145

[9] Fantasia HC. Low-dose aspirin for the prevention of preeclampsia. Nurs Womens Health. (2018) 22:87–92. doi: 10.1016/j.nwh.2017.12.002 29433703

[10] Dekker GA Sibai BM. Low-dose aspirin in the prevention of preeclampsia and fetal growth retardation: rationale, mechanisms, and clinical trials. Am J Obstet Gynecol. (1993) 168:214–27. doi: 10.1016/S0002-9378(12)90917-5 8420330

[11] Sarma A Scott NS. Aspirin use in women: current perspectives and future directions. Curr Atheroscler Rep. (2016) 18:74. doi: 10.1007/s11883-016-0630-1 27807733

[12] Lin L Huai J Li B Zhu Y Juan J Zhang M . A randomized controlled trial of low-dose aspirin for the prevention of preeclampsia in women at high risk in China. Am J Obstet Gynecol. (2022) 226:251.e1–e12. doi: 10.1016/j.ajog.2021.08.004 34389292

[13] Singh K Lia M Prakasan Sheeja A Federbusch M Gupta A Elwakiel A . Increased platelet activation and thrombo-inflammation in early and late-onset preeclampsia. Res Pract Thromb Haemost. (2025) 9:102956. doi: 10.1016/j.rpth.2025.102956 40746439 PMC12312021

[14] Nielsen HL Kristensen SD Thygesen SS Mortensen J Pedersen SB Grove EL . Aspirin response evaluated by the VerifyNow^TM^ aspirin system and light transmission aggregometry. Thromb Res. (2008) 123:267–73. doi: 10.1016/j.thromres.2008.03.023 18499236

[15] Warner TD Nylander S Whatling C. Anti-platelet therapy: cyclo-oxygenase inhibition and the use of aspirin with particular regard to dual anti-platelet therapy. Br J Clin Pharmacol. (2011) 72:619–33. doi: 10.1111/j.1365-2125.2011.03943.x 21320154 PMC3195738

[16] Walsh S. Low-dose aspirin: treatment for the imbalance of increased thromboxane and decreased prostacyclin in preeclampsia. Am J Perinatol. (1989) 6:124–32. doi: 10.1055/s-2007-999562 2653334

[17] Moore GS Allshouse AA Post AL Galan HL Heyborne KD. Early initiation of low-dose aspirin for reduction in preeclampsia risk in high-risk women: a secondary analysis of the MFMU high-risk aspirin study. J Perinatol. (2015) 35:328–31. doi: 10.1038/jp.2014.214 25474553 PMC4838902

[18] Tousty P Fraszczyk-Tousty M Dzidek S Jasiak-Józwik H Michalczyk K Kwiatkowska E . Low-dose aspirin after ASPRE—more questions than answers? Current international approach after PE screening in the first trimester. Biomedicines. (2023) 11:1495. doi: 10.3390/biomedicines11061495 37371598 PMC10295279

[19] Roberge S Villa P Nicolaides K Giguère Y Vainio M Bakthi A . Early administration of low-dose aspirin for the prevention of preterm and term preeclampsia: a systematic review and meta-analysis. Fetal Diagn Ther. (2012) 31:141–6. doi: 10.1159/000336662 22441437

[20] Zhu J Huang R Zhang J Ye W Zhang J. A prophylactic low-dose aspirin earlier than 12 weeks until delivery should be considered to prevent preeclampsia. Med Hypotheses. (2018) 121:127–30. doi: 10.1016/j.mehy.2018.08.005 30396465

[21] Banala C Moreno S Cruz Y Boelig RC Saccone G Berghella V . Impact of the ACOG guideline regarding low-dose aspirin for prevention of superimposed preeclampsia in women with chronic hypertension. Am J Obstet Gynecol. (2020) 223:419.e1–e16. doi: 10.1016/j.ajog.2020.03.004 32173446 PMC8299295

[22] Woldeamanuel GG Tlaye KG Wang X Nguyen-Hoang L Zhou Q Wang Y . Platelets in preeclampsia: an observational study of indices associated with aspirin nonresponsiveness, activation and transcriptional landscape. BMC Med. (2025) 23:346. doi: 10.1186/s12916-025-04132-9 40490753 PMC12150514

[23] Boelig RC Foecke Munden E Zhan T McKenzie SE Kraft WK. Pharmacodynamics of aspirin through gestation: predictors of aspirin response and association with pregnancy outcome, a prospective cohort study. Clin Transl Sci. (2025) 18:e70167. doi: 10.1111/cts.70167 40040304 PMC11880114

[24] Kupka E Hesselman S Gunnarsdóttir J Wikström AK Cluver C Tong S . Prophylactic aspirin dose and preeclampsia. JAMA Netw Open. (2025) 8:e2457828. doi: 10.1001/jamanetworkopen.2024.57828 39899294 PMC11791696

[25] Pennington KA Schlitt JM Jackson DL Schulz LC Schust DJ. Preeclampsia: multiple approaches for a multifactorial disease. Dis Model Mech. (2012) 5:9–18. doi: 10.1242/dmm.008516 22228789 PMC3255538

[26] Loussert L Vidal F Parant O Hamdi SM Vayssiere C Guerby P. Aspirin for prevention of preeclampsia and fetal growth restriction. Prenat Diagn. (2020) 40:519–27. doi: 10.1002/pd.5645 31955436

[27] Michita RT Kaminski V de L Chies JAB. Genetic variants in preeclampsia: lessons from studies in latin-american populations. Front Physiol. (2018) 9:1771. doi: 10.3389/fphys.2018.01771 30618791 PMC6302048

[28] Riano-Moreno JC Vargas-Castellanos E Pedraza A Díaz-Quiñonez LS Rangel-Ramos VS. Preeclampsia prediction and diagnosis: a comprehensive historical review from clinical insights to omics perspectives. Front Med. (2025) 12:1689745. doi: 10.3389/fmed.2025.1689745 41210849 PMC12588922

[29] Zhuang B Shang J Yao Y HLA-G. An important mediator of maternal-fetal immune-tolerance. Front Immunol. (2021) 12:744324. doi: 10.3389/fimmu.2021.744324 34777357 PMC8586502

[30] Ji S Xin H Li Y Su EJ. FMS-like tyrosine kinase 1 (FLT1) is a key regulator of fetoplacental endothelial cell migration and angiogenesis. Placenta. (2018) 70:7–14. doi: 10.1016/j.placenta.2018.08.004 30316329 PMC6342273

[31] Dawidowicz M Kula A Swietochowski P Ostrowska Z. Assessment of the impact of PTGS1, PTGS2 and CYP2C9 polymorphisms on pain, effectiveness and safety of NSAID therapies. Postepy Hig Med Dośw. (2020) 74:504–16. doi: 10.5604/01.3001.0014.5497

[32] Silva GFD Lopes BM Moser V Ferreira LE. Impact of pharmacogenetics on aspirin resistance: a systematic review. Arq Neuropsiquiatr. (2023) 81:062–73. doi: 10.1055/s-0042-1758445 36918009 PMC10014202

[33] Sun Z Wu Y Liu S Hu S Zhao B Li P . Effects of Panax Notoginseng Saponins on esterases responsible for aspirin hydrolysis *in vitro*. Int J Mol Sci. (2018) 19:3144. doi: 10.3390/ijms19103144 30322078 PMC6213075

[34] Jourdi G Lordkipanidzé M Philippe A Bachelot-Loza C Gaussem P. Current and novel antiplatelet therapies for the treatment of cardiovascular diseases. Int J Mol Sci. (2021) 22:13079. doi: 10.3390/ijms222313079 34884884 PMC8658271

[35] Alipova G Ablakimova N Tussupkaliyeva K Bermagambetova S Kosmuratova S Karimsakova B . Prevention of pre-eclampsia: modern strategies and the role of early screening. J Clin Med. (2025) 14:2970. doi: 10.3390/jcm14092970 40364001 PMC12072587

[36] Regan CL McAdam BF McParland P Boylan PC FitzGerald GA Fitzgerald DJ. Reduced fetal exposure to aspirin using a novel controlled-release preparation in normotensive and hypertensive pregnancies. BJOG Int J Obstet Gynaecol. (1998) 105:732–8. doi: 10.1111/j.1471-0528.1998.tb10203.x 9692413

[37] Pai CH Yen CT Chen CP Yu IS Lin SW Lin SR. Lack of thromboxane synthase prevents hypertension and fetal growth restriction after high salt treatment during pregnancy. PLoS ONE. (2016) 11:e0151617. doi: 10.1371/journal.pone.0151617 26974824 PMC4790927

[38] Xu ZH Jiao JR Yang R Luo BY Wang XF Wu F. Aspirin resistance: clinical significance and genetic polymorphism. J Int Med Res. (2012) 40:282–92. doi: 10.1177/147323001204000128 22429367

[39] Chaudhary R Bliden KP Garg J Mohammed N Tantry U Mathew D . Statin therapy and inflammation in patients with diabetes treated with high dose aspirin. J Diabetes Complications. (2016) 30:1365–70. doi: 10.1016/j.jdiacomp.2016.05.002 27237049

[40] Udeh PI Olumodeji AM Kuye-Kuku TO Orekoya OO Ayanbode O Fabamwo AO. Evaluating mean platelet volume and platelet distribution width as predictors of early-onset pre-eclampsia: a prospective cohort study. Matern Health Neonatol Perinatol. (2024) 10:5. doi: 10.1186/s40748-024-00174-8 38424566 PMC10905831

[41] Karateke A Kurt R Baloglu A. Relation of platelet distribution width (PDW) and platelet crit (PCT) to preeclampsia. Pol Gynaecol. (2015) 86:372–5. doi: 10.17772/gp/2425 26117976

[42] Bashyal R Singh A Maharjan S Tuladhar S Bhattarai B Sharma PK. Platelet count-to-platelet distribution width ratio and other platelet indices as cost-effective markers of preeclampsia: a case control study. Kathmandu Univ Med J KUMJ. (2024) 22:367–72. doi: 10.21203/rs.3.rs-3833364/v1 40457903

[43] AlSheeha MA Alaboudi RS Alghasham MA Iqbal J Adam I. Platelet count and platelet indices in women with preeclampsia. Vasc Health Risk Manag. (2016) 12:477–80. doi: 10.2147/VHRM.S120944 27920548 PMC5123587

[44] Lordkipanidze M Pharand C Schampaert E Turgeon J Palisaitis DA Diodati JG . comparison of six major platelet function tests to determine the prevalence of aspirin resistance in patients with stable coronary artery disease. Eur Heart J. (2007) 28:1702–8. doi: 10.1093/eurheartj/ehm226 17569678

[45] Shantsila E Kamphuisen PW Lip GYH. Circulating microparticles in cardiovascular disease: implications for atherogenesis and atherothrombosis. J Thromb Haemost. (2010) 8:2358–68. doi: 10.1111/j.1538-7836.2010.04007.x 20695980

[46] Rădulescu C Bacârea A Huţanu A Gabor R Dobreanu M. Placental growth factor, soluble fms-like tyrosine kinase 1, soluble endoglin, IL-6, and IL-16 as biomarkers in preeclampsia. Mediators Inflamm. (2016) 2016:3027363. doi: 10.1155/2016/3027363 27799724 PMC5069373

[47] Kusanovic JP Romero R Chaiworapongsa T Erez O Mittal P Vaisbuch E . A prospective cohort study of the value of maternal plasma concentrations of angiogenic and anti-angiogenic factors in early pregnancy and midtrimester in the identification of patients destined to develop preeclampsia. J Matern Fetal Neonatal Med. (2009) 22:1021–38. doi: 10.3109/14767050902994754 19900040 PMC3427777

[48] Hernandez I Chissey A Guibourdenche J Atasoy R Coumoul X Fournier T . Human placental NADPH oxidase mediates sFlt-1 and PlGF secretion in early pregnancy: exploration of the TGF-β1/p38 MAPK pathways. Antioxid Basel Switz. (2021) 10:281. doi: 10.3390/antiox10020281 33673360 PMC7918586

[49] Lv P Lu LF. Diagnostic value of sFlt-1/PlGF-1 ratio and plasma PROK1 for adverse pregnancy outcomes in women with hypertensive disease of pregnancy. Kaohsiung J Med Sci. (2024) 40:1068–76. doi: 10.1002/kjm2.12907 39625119 PMC11618490

[50] Jacobson RL Brewer A Eis A Siddiqi TA Myatt L. Transfer of aspirin across the perfused human placental cotyledon. Am J Obstet Gynecol. (1991) 165:939–44. doi: 10.1016/0002-9378(91)90444-V 1951559

[51] Ahn TG Hwang JY. Preeclampsia and aspirin. Obstet Gynecol Sci. (2023) 66:120–32. doi: 10.5468/ogs.22261 36924072 PMC10191759

[52] Liu M Wang RB Xing JH Tang YX. Nested case-control study of corin combined with sFlt-1/PLGF in predicting the risk of preeclampsia. Int J Gen Med. (2021) 14:2313–20. doi: 10.2147/IJGM.S297344 34113161 PMC8184237

[53] Cofer LB Barrett TJ Berger JS. Aspirin for the primary prevention of cardiovascular disease: time for a platelet-guided approach. Arterioscler Thromb Vasc Biol. (2022) 42:1207–16. doi: 10.1161/ATVBAHA.122.318020 36047408 PMC9484763

[54] Moore GS Allshouse AA Winn VD Galan HL Heyborne KD. Baseline placental growth factor levels for the prediction of benefit from early aspirin prophylaxis for preeclampsia prevention. Pregnancy Hypertens. (2015) 5:280–6. doi: 10.1016/j.preghy.2015.06.001 26597741 PMC4841270

[55] Wang Y Wang L Yu X Gong W. MiR-30a-3p targeting FLT1 modulates trophoblast cell proliferation in the pathogenesis of preeclampsia. Horm Metab Res. (2022) 54:633–40. doi: 10.1055/a-1880-1126 35981547

[56] Saxena U Lachyan A Debnath A Gupta S Yadav A Kishore J . Effectiveness of low-dose aspirin (75–150 mg) in preventing preeclampsia among high-risk pregnant women: a systematic review and meta-analysis of randomized controlled trials. Obstet Gynecol. (2025) doi: 10.1101/2025.03.27.25324291

[57] Hernandez F Chavez H Goemans SL Kirakosyan Y Luevano CD Canfield D . Aspirin resistance in pregnancy is associated with reduced interleukin-2 (IL-2) concentrations in maternal serum: implications for aspirin prophylaxis for preeclampsia. Pregnancy Hypertens. (2024) 37:101131. doi: 10.1016/j.preghy.2024.101131 38851168 PMC11610477

[58] Rk K Ramakrishnan KK Gunasekaran D Aram A Natarajan P. Role of uterine artery doppler study between 11 and 14 weeks as a predictor of preeclampsia. Cureus. (2024) 16:e63591. doi: 10.7759/cureus.63591 39087160 PMC11290376

[59] Mallampati D Grobman W Rouse DJ Werner EF. Strategies for prescribing aspirin to prevent preeclampsia: a cost-effectiveness analysis. Obstet Gynecol. (2019) 134:537–44. doi: 10.1097/AOG.0000000000003413 31403606

[60] Grünebaum A McCullough LB Litvak A Chervenak FA. Inclusion of pregnant individuals among priority populations for coronavirus disease 2019 vaccination for all 50 states in the United States. Am J Obstet Gynecol. (2021) 224:536–9. doi: 10.1016/j.ajog.2021.01.026 33545113 PMC7906826

[61] Atallah A Lecarpentier E Goffinet F Doret-Dion M Gaucherand P Tsatsaris V. Aspirin for prevention of preeclampsia. Drugs. (2017) 77:1819–31. doi: 10.1007/s40265-017-0823-0 29039130 PMC5681618

[62] Ray JG Abdulaziz KE Berger H DOH-NET (Diabetes Obesity and Hypertension in Pregnancy Research Network). Aspirin use for preeclampsia prevention among women with prepregnancy diabetes, obesity, and hypertension. JAMA. (2022) 327:388–90. doi: 10.1001/jama.2021.22749 35076678 PMC8790661

[63] Boelig RC Wanees M Zhan T Berghella V Roman A. Improving utilization of aspirin for prevention of preeclampsia in a high-risk urban cohort: a prospective cohort study. Am J Perinatol. (2021) 38:544–52. doi: 10.1055/s-0040-1718580 33099285 PMC8491097

[64] Nguyen-Hoang L Sahota DS Tai AST Chen Y Feng Q Wang X . Effect of aspirin on biomarker profile in women at high risk for preeclampsia. Am J Obstet Gynecol. (2025) 232:561.e1–e20. doi: 10.1016/j.ajog.2024.11.007 39547345

[65] Kumasawa K Kashiwabara K Inoue M Kaneko K Hyodo H Yamashita T . Pravastatin for the prevention of recurrent hypertensive disorders of pregnancy: study protocol for a randomized, open-label, parallel-group, three-arm trial. Trials. (2025) 26:499. doi: 10.1186/s13063-025-09136-7 41225548 PMC12613540

[66] Chirilă CN Mărginean C Ghiga DV Voidăzan S Chirilă PM Gliga ML . Second trimester prediction algorithm for early-onset hypertensive disorders of pregnancy occurrence and severity based on soluble fms-like tyrosine kinase 1 (sFlt-1)/placental growth factor (PlGF) ratio and uterine doppler ultrasound in women at risk. Child Basel Switz. (2024) 11:468. doi: 10.3390/children11040468 PMC11049313 38671685

